# Ten years of experience with the Ponto bone‐anchored hearing system—A systematic literature review

**DOI:** 10.1111/coa.13556

**Published:** 2020-05-25

**Authors:** Helén Lagerkvist, Karin Carvalho, Marcus Holmberg, Ulrika Petersson, Cor Cremers, Malou Hultcrantz

**Affiliations:** ^1^ Oticon Medical AB Askim Sweden; ^2^ Oticon Medical Smørum Denmark; ^3^ Department of Otorhinolaryngology Donders Institute for Brain, Cognition and Behaviour Radboud University Medical Centre Nijmegen Nijmegen The Netherlands; ^4^ Karolinska Institutet Stockholm Sweden

**Keywords:** BAHA, BAHS, bone conduction, mixed conductive‐sensorineural, quality of life, SSD, treatment outcome

## Abstract

**Background:**

Bone‐anchored hearing systems (BAHSs) are widely used for hearing rehabilitation and are indicated in cases of conductive and mixed hearing loss and in single‐sided deafness. The Ponto system, that is one available option, has been on the market since 2009.

**Objective of review:**

The aim of this study is to systematically review the literature reporting on the Ponto system, with regard to audiological and surgical outcomes and patient's quality‐of‐life scores.

**Type of review:**

A systematic literature search was performed in the PubMed database 2009‐July 2019.

**Search strategy:**

Search term: ((osseointegrated hearing aid) OR (bone conduction implant) OR (bone anchored hearing) OR BAHA OR BAHS OR BAHI). Pre‐defined inclusion and exclusion criteria were applied.

**Evaluation method:**

English‐language articles reporting original clinical data (audiological, surgical or quality‐of‐life outcomes) on the Ponto system were included.

Articles reporting on Ponto and another BAHS system where the results on Ponto constituted less than 50% of the patient population or including only results on testband or softband devices were excluded.

**Results:**

Audiological outcomes were discussed in 20 publications. Improvement against the unaided thresholds was demonstrated. The functional improvement was on average 33.9 dB. The effective gain or remaining air‐bone gap was on average 6.7 dB. All evaluated data showed aided speech reception thresholds significantly below normal speech level. Twenty‐seven publications reported surgical and follow‐up data for the Ponto system. Implant survival was 97.7%, adverse skin reactions (Holgers ≥ 2) were 5% across visits and 15% across patients. No complications were life‐threatening, causing permanent disability/damage or requiring a hospitalisation. Five studies reported quality of life using the Glasgow benefit inventory, 98% reported an improvement when analysing the score on an individual level.

**Conclusions:**

The outcomes of this systematic review confirm that percutaneous systems provide consistent audiological benefits and improved quality of life for patients. Further, the review demonstrates that the percutaneous systems are safe, with relatively low complication rates. Skin‐related complications are the most common complication type and are experienced by approximately one patient out of seven, or in less than one of 20 follow‐up visits.

**Key points**

- The functional improvement, the improved hearing experience by the patient, was significant and on average 33.9 dB.
- All evaluated data showed aided speech reception thresholds significantly below normal speech level.
- Approximately one of seven patients experienced adverse skin reactions among the studies included in this review.
- The survival rate of Ponto implants was 98%, including traumatic losses, over an accumulated follow‐up time of 1623 years.
- A total of 98% of the patients reported an improvement in quality of life using the Ponto system.

## 1 INTRODUCTION

Bone‐anchored hearing systems (BAHSs) are widely used for hearing rehabilitation and are indicated in cases of conductive and mixed hearing loss and in cases of single‐sided deafness (SSD).

Bone‐anchored hearing systems are based on bone conduction (BC) of sound, in which vibrations of the skull are transferred to the inner ear and transformed to a hearing sensation.^1^ The discovery that bone can firmly attach to titanium, a process termed osseointegration,^2^ further led to the development of a hearing aid attached to an osseointegrated and skin‐penetrating implant in the skull bone in the late 1970s.^3^ The development of this percutaneous BAHS eliminated the dampening effect of the skin, while preserving the high frequencies^4^ and reducing the discomfort caused by the pressure on the skin needed in conventional BC systems.

Currently, more than 200 000 patients around the world have been treated with percutaneous BAHS. The long‐term success rate of BAHS is high, with a low rate of major complications (eg Dun et al^5^ and Kiringoda and Lustig^6^ and proven patient benefits in terms of hearing rehabilitation^7^).

There are currently two percutaneous systems available on the market, the BAHA^®^ Connect (Cochlear BAS) and Ponto (Oticon Medical AB) systems. The two systems are built on the same principle: an osseointegrated screw (implant) in the temporal bone, a skin‐penetrating abutment and an external sound processor.

The purpose of this article is to present a systematic review of the literature regarding the clinical outcomes related to surgery and aftercare, audiology and quality of life with the Ponto system. Ponto has been available for 10 years,^8^ and the results over this whole period were included in the review. Thus, different generations of Ponto sound processors and implants, as well as different surgical techniques, are included in the reviewed data.

## 2 METHODS

A systematic literature search was performed in the PubMed database from 2009 (the year Ponto was released) to July 2019. The following search terms were used: “osseointegrated hearing aid” OR “BC implant” OR “bone anchored hearing” OR “BAHA” OR “BAHS” OR “BAHI.” All identified abstracts were reviewed for relevance, and full‐text articles were further reviewed and were included or excluded after applying the following criteria:

1. Inclusion criteria: Any English‐language article reporting original clinical data on the Ponto system and included at least one of the following data points: surgical, audiological or quality‐of‐life outcomes.
2. Exclusion criteria: Preclinical, cadaveric or laboratory studies and review articles; articles reporting on Ponto and another BAHS system where the results on Ponto constituted less than 50% of the patient population and studies including only results on testband or softband devices.

The included publications were divided into two groups:

1. Publications exclusively reporting on the Ponto system (and in the case of sound processor studies, data reported separately on all patients using the Ponto sound processor).
2. Publications with mixed brands of implants/sound processors where the number of Ponto implants/sound processors was specified and accounted for more than 50% of the total.

Double reporting was avoided as far as possible, and articles reporting on preliminary results for which later publications described the same cohort of patients were included in the groups (Tables 1 and 2) but not included in the meta‐analyses.

**TABLE 1:** Publications with audiological or quality of life outcomes including Ponto sound processors

Ref.	Author (year)	Study design	Patients	Ponto sound processor model	Clinical condition	Threshold outcomes	Speech in quiet	Speech in noise	Other	Quality of life
*Publications where all patients received Ponto sound processors*										
33	den Besten et al. (2016)	PC	50	Ponto Pro Ponto Pro Power Ponto Plus Ponto Plus Power	CHL, MHL SSD				x	x
12	Bianchi et al. (2019)	PC	21	Ponto Pro Ponto 3 Ponto 3 SuperPower	CHL, MHL				x	
13	Bosman et al. (2018)	PC	18	Ponto 3 SuperPower	MHL	AT: 38.3^a^ FG: 38.1^a^ EG: 3.9^a^		x	x	
				Ponto Pro Power		AT: 40.7^a^ FG: 35.7^a^ EG: 2.2^a^		x	x	
14	Bosman et al. (2016)^b^	PC	19	Ponto Pro Ponto Plus	CHL, MHL			x	x	
15	Bosman et al. (2014)	PC	19	Ponto Pro Ponto Plus	CHL, MHL			x	x	
16	Bosman et al. (2013)	PC	18	Ponto Pro Power	MHL	AT: 39.5 FG: 34.4^a^ EG: 5.3^a^	x	x	x	
17	Busch et al. (2015)	PC	11	Ponto Pro Power	CHL, MHL	AT: 32.4^a^ FG: 29 EG: 11.1^a^	x	x	x	
24	Caruso et al. (2017)	PC	20	Ponto Pro Ponto Pro Power Ponto Plus Ponto Plus Power	CHL, MHL	AT: 45 FG: 33 EG: 14^a^	x			x
25	Celikgun and Kalcioglu (2017)	PC	5	Ponto Pro Ponto Pro Power	CHL, MHL	AT: 16 FG: 36 EG: 6	x			
18	Hill‐Feltham et al. (2014)	PC	14	Ponto Pro	CHL, MHL			x	x	
28	Lunner et al. (2016)	PC	16	Ponto Plus Power	CHL, MHL				x	
35	Nelissen et al. (2016)	RCT	57	Unknown	CHL, MHL SSD				x	x
47	Nelissen et al. (2013)	R	31	Unknown	CHL, MHL SSD		x			x
21	Oeding and Valente (2013)	PC	15	Ponto Pro	SSD			x	x	
19	Olsen et al. (2011)	PC	12	Ponto Pro	CHL, MHL SSD	AT: 31		x	x	
27	Pittman (2019)	PC	17	Ponto Plus Power	CHL SSD				x	
26	Rigato et al. (2016)	PC	6	Ponto Pro Power	CHL, MHL	FG: 32	x	x	x	x
23	Wang et al. (2018)	RC	6	Ponto Pro	CHL	AT: 18^c^ FG: 42^c^ EG: 0^c^	x		x	
*Publications with mixed sound processors, ≥50% Ponto*										
22	Finbow et al. (2015)	PC	8	Ponto Pro	SSD		x	x	x	
20	Kara et al. (2019)	PC	20	Ponto Plus	CHL, MHL	AT: 38 EG: 11	x	x		

AT, average aided threshold; CR, case report; EG, average effective gain; FG, average functional gain; P, prospective; PC, prospective controlled; R, retrospective; RC, retrospective controlled; RCT, randomized controlled trial.

^a^ Value based on personal communication with authors.

^b^ The same study (but other outcomes) reported in Bosman,^15^ patients therefore not included in total.

^c^ Average of all measured frequencies instead of PTA4 (average of 0.5, 1, 2 and 4 kHz).

**TABLE 2:** Publications with surgical outcomes including Ponto implants

Ref.	Author	Study design	Patients (Implants)	Ponto implant model	Surgical method	Follow‐up time (mo)	Implant survival^e^ (%)	Holgers ≥ 2
*Publications with only Ponto implants*								
32	Calon et al. (2018)	RCT	63 (63)	Wide Ponto	TP, MIPS	3	92.1	13% across pat. (8/63)
24	Caruso et al. (2016)	R	49 (49)	Wide Ponto	TP	9‐20	100.0	4% across visits (5/121)
33	den Besten et al. (2016)^a^	PC	25 (25)	Wide Ponto	TP	6	100.0	28% across pat. (7/25)
48	Foghsgaard et al. (2014)	P	20 (20)	Wide Ponto	TR	11.5‐15.3, m 12.6	100.0	3% across visits (2/76)
49	Hultcrantz (2015)^b^	CR	2 (4)	Wide Ponto	TP	12	50.0	—
50	Johansson et al. (2017)	SE	76(77)	Wide Ponto	MIPS	5‐9.8, m 8.5	96.1	5% across visits (8/160) 9.2% across pat. (7/76)
51	Kim et al. (2019)	R	75 (75)	Wide Ponto, Ponto BHX	MIPS	0.25‐2.25	98.7	7% across visits (10/143) 6% across pat. (4/70)
34	Kruyt (2019)	PC	25 (25)	Wide Ponto	TP	36	100.0	36% across pat. (9/25)
34, 52	Kruyt et al. (2018)^a^	RCT	57 (59)	Ponto 3.75, Wide Ponto	TR	36	98.3	17% across pat. (10/59)
53	Kruyt et al. (2018)	R	34 (34)	Ponto BHX	TR, TP	7‐17, m 15	97.0	12% across pat. (4/34)
54	Mowinckel et al. (2016)	P	24 (24)	Wide Ponto	TP	12	100.0	8% across visits (7/90) 17% across pat. (4/24)
55	Muzaffar et al. (2014)	P	15 (20)	Ponto 3.75	TR	0.5‐2.25	95.0	0%
47	Nelissen et al. (2013)	R	31 (31)	Ponto 3.75	TR	12.1‐25.2	96.8	4% across visits (4/94)
35, 36	Nelissen et al. (2015)^c^	RCT	57 (59)	Ponto 3.75, Wide Ponto	TR	6	100.0	3% across visits 8% across implants (5/59)
57	Reznitsky et al. (2018)	P	48 (48)	Wide Ponto	TR, TP	48‐60	98.0	4% across visits (14/326) 195 across pat. (9/48)
58	Sardiwalla et al. (2018)	R	12 (12)	Wide Ponto, Ponto BHX	Punch	22	100.0	—
59	Trobos et al. (2018)^d^	P	7 (7)	Wide Ponto	TP	12	100.0	0
60	Wazen et al. (2016)	P	30 (30)	Wide Ponto	TP	12	100.0	1% across visits (1/180) 3% across pat. (1/30)
61	Westover et al. (2018)	P	39 (39)	Ponto 3.75, Ponto BHX	TP, MIPS	11.6‐13.3 m 12.4	100.0	—
*Publications with mixed implants, ≥50% Ponto*								
62	Di Giustino et al. (2018)	RC	29 (30)	Mix (60% Ponto)	TR, TP, MIPS	12	93.3	4% across visits (5/119)
63	Dumon et al. (2015)	PC	40 (40)	Mix (55% Ponto)	TR, Punch	6‐18, m 10.5	97.5	14% across visits (14/99)
64	Goldman et al. (2013)	R	14 (15)	Mix (67% Ponto)	TP, Punch	9‐20, m 14.8	100.0	—
65	Gordon et al. (2015)	RC	51 (51)	Mix (70% Ponto)	TR, Punch	0.25‐25	99.0	8% across pat. (8/102)
66	Hultcrantz et al. (2015)	P	17 (17)	Mix (59% Ponto)	TP	12	100	6% across pat. (1/17)
67	Singam et al. (2014)	R	30 (30)	Mix (73% Ponto)	TP	6‐42, m 23	100.0	—
68	Strijbos et al. (2016)	RC	203 (211)	Mix (51% Ponto)	TR	11.2‐35.3	98.5	8% across pat. (34/211)
69	Wise et al. (2018)	RC	130 (130)	Mix (58% Ponto)	TR	6‐29, m 16.4	97.7	21% across pat. (27/130)

Abbreviations: CR, case report; MIPS, Minimally Invasive Ponto surgery; P, prospective; PC, prospective controlled; R, retrospective; RC, retrospective controlled; RCT, randomized controlled trial; TP, tissue preservation; TP, tissue preservation; TR, tissue reduction.

^a^ Control group not included due to duplication reports (Nelissen, 2015).

^b^ Paediatric patients only.

^c^ 3 y data published,^34^ thus not included in meta‐analyses.

^d^ Investigational non‐commercial device excluded.

^e^ Not including elective removal.

### 2.1 Audiological outcome measures

All audiological outcomes reported in the publications were categorised into four groups: threshold‐based, speech in quiet, speech in noise and other.

For the threshold‐based measures, meta‐analyses were performed for functional gain (the difference between unaided and aided sound‐field thresholds) and effective gain.^9^, ^10^ The effective gain/BC gain (or remaining air‐bone gap) is calculated as the difference between the aided sound‐field threshold and the BC threshold. Random effect models using the restricted maximum‐likelihood method were fitted using JASP (University of Amsterdam, Amsterdam, The Netherlands, version 0.11.0.1). A heterogeneity test was performed, and Q and *I* ^2^ statistics were reported.

The results from the speech intelligibility test, both in quiet and in background noise, are summarised in the tables.

### 2.2 Surgical/medical outcome measures

Implant survival and adverse skin reactions according to Holgers classification^11^ (Holgers ≥ 2) were investigated, and a meta‐analysis was performed. In addition, the intra‐operative events and postsurgical complications reported in the publications were summarised.

#### 2.2.1 Patient satisfaction measures

Quality‐of‐life data, but no other self‐reported outcomes, were included in the review.

#### 2.2.2 Level of evidence

As part of the review, the study designs were collected, no limitation on the level of evidence was applied.

#### 2.2.3 Ethical considerations

No ethical considerations were made as this is a review of existing literature.

## 3 RESULTS

**FIGURE 1:**
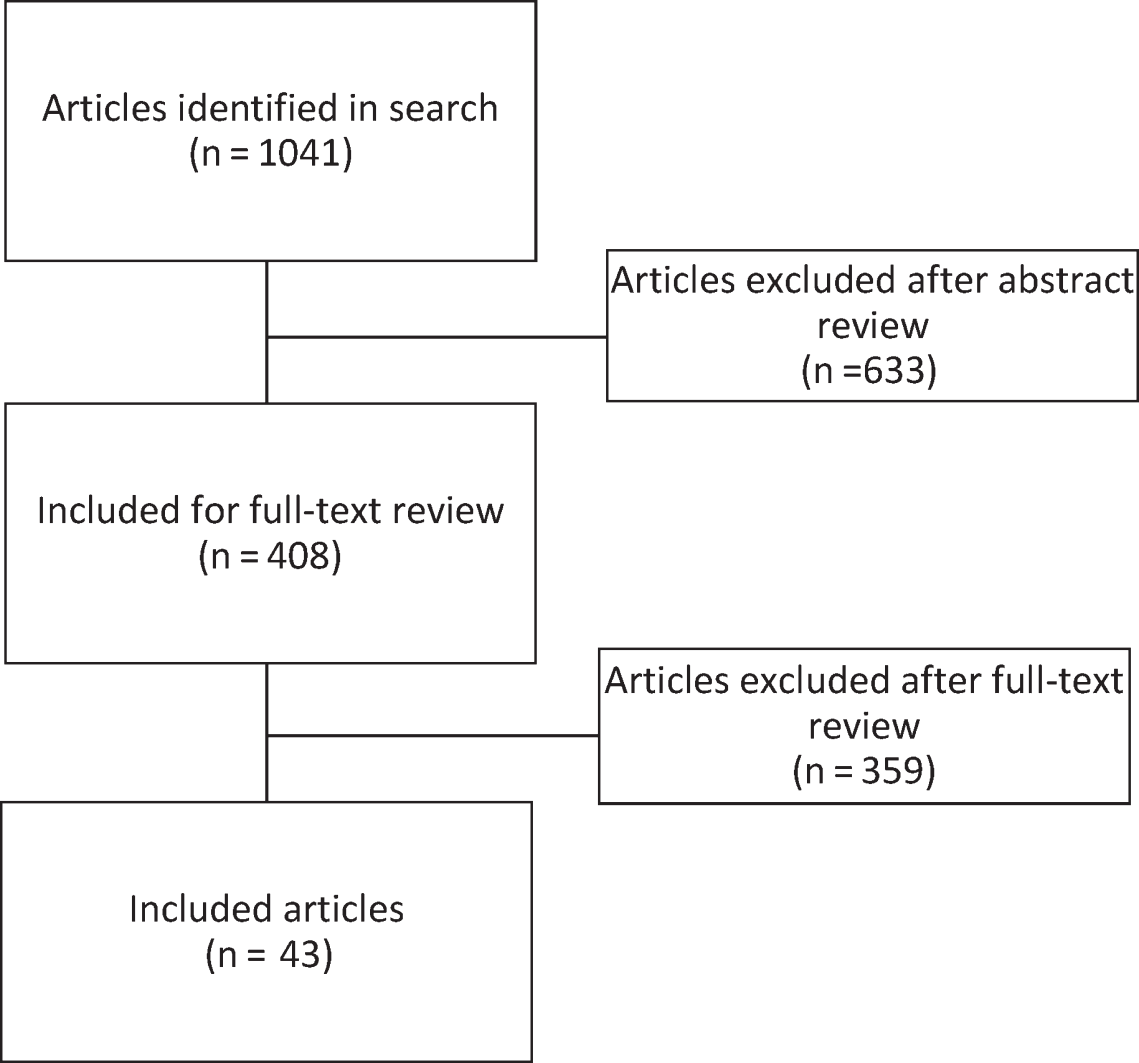
Flow chart of the systematic literature review

The search strategy yielded 1041 publications (Figure 1). After reviewing the abstracts, 408 publications were selected for further full‐text review. After the full‐text review, 68 publications mentioned the use of Ponto. Forty‐three publications (41 studies) that fulfilled the inclusion criteria were used for the analyses.

The results of the literature search are summarised in two tables; Table 1 lists all included publications with audiological or quality‐of‐life outcomes, including the main findings. Table 2 lists all included publications with intra‐ and postoperative results on the implant system. Four publications are repeated in both tables.

Two studies were randomised controlled studies (Tables 1 and 2). The majority of studies were prospective or retrospective controlled studies (22/41, 18 and four, respectively), eight were prospective and seven retrospective without control, and two publications were case reports. The average number of patients in the audiological studies was 19 and in the surgical studies 44.

### 3.1 Audiological outcomes with the Ponto system

Table 1 summarises the publications that reported audiological outcomes. A total of 20 publications (19 studies) with 364 patients were included (Group A: 18 publications, 336 patients; Group B: two publications, 14 Ponto patients out of a total of 28 patients).

Several different models of the Ponto sound processors were used in the reviewed studies and are listed in Table 1: Ponto Pro was launched in 2009; Ponto Pro Power in 2011; Ponto Plus and Ponto Plus Power in 2013; and Ponto 3, Ponto 3 Power and Ponto 3 Superpower in 2016.

Within‐subject comparisons of different generations of Ponto sound processors were performed in three studies.^12^, ^13^, ^14^, ^15^ Comparisons to other brands of sound processors were performed with a within‐subject crossover design in four studies^16^, ^17^, ^18^, ^19^ and between groups in two studies.^20^, ^21^ Comparison to other treatment options was performed for SSD patients and contralateral routing of signals devices with a within‐subject design.^22^ A single study reported the difference between patients with softband and a subset of patients with implanted devices.^23^ Studies with only softband results were excluded. For this review, we were interested in the outcomes across Ponto sound processors.

**FIGURE 2:**
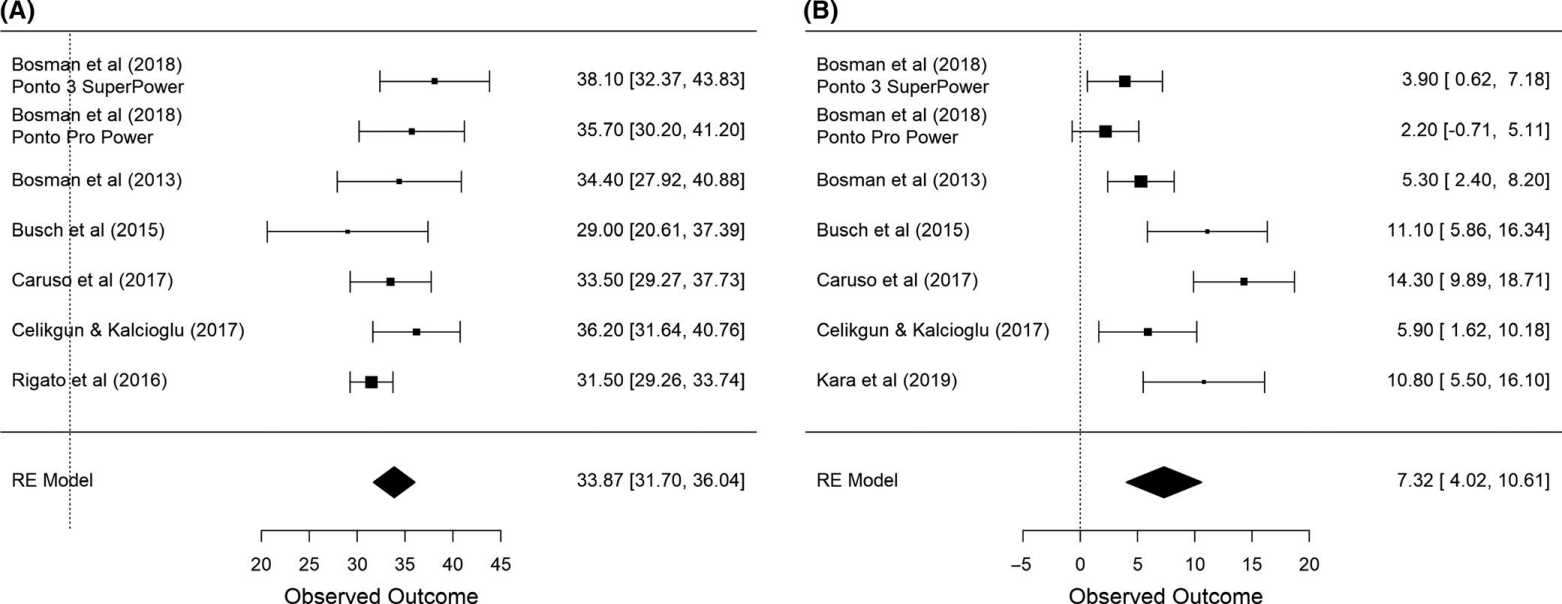
A, Functional gain in the reviewed papers. B, Effective gain. Tree plots reflect studies reporting both average and standard deviations of the outcome measure. Below the line, the weighted average of the meta‐analyses is displayed.

Functional gain was reported in seven studies.^13^, ^16^, ^17^, ^23^, ^24^, ^25^, ^26^ No study reported effective gain; however, the (average) effective gain for Ponto devices could be derived from seven studies.^13^, ^16^, ^17^, ^20^, ^23^, ^24^, ^25^ Six studies also reported standard deviations or individual data for respective outcome, allowing these data to be included in the meta‐analysis models (Figure 2). Speech recognition results in quiet were reported in nine studies (speech reception threshold [SRT] and/or percentage correct at a fixed level), and 11 studies reported speech in noise results (speech recognition scores in fixed background noise or adaptive signal‐to‐noise ratio thresholds).

Table 3 summarises the results for aided threshold‐based results. The table presents the average data reported per study. The average aided thresholds reported per study varied between 16 and 45 dB in hearing level (HL) (4‐pure tone average [PTA4], an average of 0.5, 1, 2 and 4 kHz in all studies except one^23^ where the PTA was calculated across all measured frequencies). The average aided threshold was 33.1 dB HL. This value can be compared to a normal‐hearing threshold that is defined as equally or better than 25 dB HL.

**TABLE 3:** Summary of hearing loss and aided thresholds (average values reported per study were used for the calculations)

Outcome	Number of studies reporting the variable	Number of patients	Value ± SD (min, max)
PTA_BC_	8	104	24.6 dB HL ± 8.8 (10, 37)
Aided threshold	8	110	33.1 dB HL ± 10.2 (16, 45)
Functional gain	7	84	35.1 dB ± 4.0 (29, 42)
Effective gain	7	98	6.7 dB ± 4.9 (0, 14)

Functional gain, the improvement compared to the unaided condition, was significant in all studies that reported this measure.^13^, ^16^, ^17^, ^23^, ^24^, ^25^, ^26^

The overall weighted functional gain from the meta‐analysis displayed in Figure 2A (six studies with seven sound processors) gave a functional gain of 33.9 dB (95% CI: 31.7, 36.0). The random effect meta‐analysis revealed non‐significant heterogeneity (*P* < .20, *Q* = 8.6, *df* = 6, *I* ^2^ = 32.9%).

The calculated average effective gain, or remaining air‐bone gap, was 6.7 dB and varied between 0 and 17 dB across studies (Table 3). The weighted effective gain from the meta‐analysis (six studies with seven sound processors) was 7.3 dB (95% CI: 4.0, 10.6). The random effect meta‐analysis indicated highly heterogeneous data (*P* < .001, *Q* = 28.7, *df* = 6, *I* ^2^ = 80.6%), and therefore, the confidence interval should be interpreted with caution (Figure 2B).

Aided thresholds were reported for predominantly conductive and mixed groups, with two SSD patients included in the data. The average BC threshold of the conductive/mixed population in the review was 24.6 dB HL, with individual studies reporting averages between 10 and 37 dB HL (Table 3). Adding BC thresholds as covariates in the random effect analysis did not significantly change the conclusion about heterogeneity of the data.

For benefits in terms of speech perception, either measured as the lowest level needed to understand speech (aided SRT) or understand speech in noise, the Ponto devices demonstrated benefits compared to unaided (summarised in Tables S6 and S7). Although speech tests are standardised for a given language, they vary due to different speech materials in different countries, different loudspeaker configurations and other factors, making direct comparisons difficult. To quantify the improvements, the aided SRTs can be compared to a normal speech level, defined as 65 dB sound pressure level (SPL). All evaluated data showed aided SRTs significantly below this level (on average 42.5 dB SPL), demonstrating the ability to understand speech with the devices under investigation at lower than normal speech levels.

Among the other outcome measures, the following were performed or assessed: listening effort assessment by means of pupillometry,^12^ the ability to perform lexical decision tasks, the ability to detect nonsense words in context, rapid word learning,^27^ memory recall by the sentence‐final Word Identification and Recall Test (SWIR) test,^28^ the Abbreviated Profile of Hearing Aid Benefit^13^, ^14^, ^15^, ^16^, ^17^, ^21^, ^26^ and the Speech Spatial and Qualities of Hearing scale.^13^, ^14^, ^15^, ^16^, ^17^, ^22^

### 3.2 Intra‐operative events and post‐surgical complications

Twenty‐seven publications (26 studies) reported surgical outcomes (1146 patients, 1166 implants), which are presented in Table 1 (Group A: 19 publications, 642 Ponto implants; Group B: eight publications, 301 Ponto implants out of 524 total).

Different models of implants were used in the reviewed studies. The first Ponto implant had a diameter of 3.75 mm. To increase the surface available for osseointegration and thus the initial stability, the Wide Ponto implant with a diameter of 4.5 mm was developed and released in 2012. In 2016, the Ponto BHX implant was introduced. The macroscopic design of the Ponto BHX implant is identical to that of the Wide Ponto implant but with a site‐specific laser‐modified surface to further enhance osseointegration.^29^ The abutment design and surface were identical throughout the studies, although longer abutments (12 and 14 mm) were added over time. Across all studies, 49% of the implants were Wide Ponto implants, 6% were the Ponto BHX type, and 12% were 3.75 mm implants. The remaining implants were unaccounted for. For group A, the corresponding numbers are 73%, 9% and 18%, respectively.

In addition, the development of surgical techniques over the 10 years covered in this review has been rapid. Across all reviewed studies, tissue reduction was used in 40% of the implant installations, whereas tissue preservation techniques, first described by Hultcrantz,^30^ including linear incision and punch only techniques, were used in 60% of the installations. In only Group A, 16% of the implants were installed using tissue reduction techniques, 52% using tissue preservation with a linear incision approach and 33% using minimally invasive Ponto surgery.

The accumulated follow‐up time for the included publications (reporting surgical and postsurgical events and complications) was 1649 years. The average follow‐up time across the studies was 15.9 months, 17 months across patients, with a range of 0.25‐60 months.

The overall survival rate over all publications in subgroups A and B (N = 1166) was 97.7%. Of the publications reporting solely on Ponto implants (N = 642), the implant survival rate was 97.5%.

Across all studies, the rates of adverse skin reactions (Holgers ≥ 2) were reported to be 5% across visits (70/1408) and 15% across patients (133/863). The corresponding overall figures for the Ponto‐only publications were 4% across visits (51/1190) and 16% across patients (63/403). It should be noted that not all publications reported Holgers classification across patients and across visits.

Table 4 lists other complications/events reported, including the rates calculated across the total number of implants. Pain and numbness were reported in several studies, but due to inconsistent reporting, no conclusion regarding the symptoms that remained at the end of the study can be made. Detailed reports of the complete set of defined outcome measures are available in Table S8.

**TABLE 4:** Complications/events reported (groups A and B)

Complication/event type	Number of studies reporting the variable	Number of implants	Observations (% of number implants)	Ref.
Dura exposure	5	294	19 (6)	24, 32, 50, 51, 62
Cerebrospinal fluid leak	5	294	1 (0.3)	24, 32, 50, 51, 62
Drilling into vein or bleeding	6	318	25 (8)	24, 32, 50, 51, 54, 62
Holgers 4	19	769	3 (0.4)	24, 33, 34, 47, 48, 50, 51, 52, 53, 54, 59, 60, 62, 63, 64, 65, 66, 67, 69
Skin revision surgery	15	773	26 (3)	24, 32, 33, 34, 47, 48, 51, 52, 53, 58, 63, 64, 66, 67, 68, 69
Haematoma	1	63	2 (3)	32
Abscess	1	130	2 (2)	69
Scar hypertrophy	1	130	1 (1)	69
Pain and numbness outcome reported	8	354	Various measures used	32, 33, 34, 50, 53, 54, 58, 66
Abutment change	12	666	27 (4)	24, 32, 33, 47, 48, 54, 64, 65, 66, 67, 68, 69
Abutment removal	6	264	5 (2)	24, 32, 34, 52, 65, 66

No complications related to the device that were life‐threatening, caused permanent disability/damage or required hospitalisation for significant duration (defined as >24 hours) were reported in the reviewed publications.

### 3.3 Quality‐of‐life outcome

Five studies with a total of 176 patients reported quality of life (Table 5). All studies used the Glasgow benefit inventory (GBI), a generic health‐related quality‐of‐life questionnaire developed specifically for otorhinolaryngological interventions.^31^ Table 5 summarises the GBI scores from the reviewed literature. The GBI uses a scale from −100 to +100, where scores above 0 indicate improved quality of life. On average, the total GBI score across studies was 32.6. The studies demonstrated the greatest improvement in the general and social subscales (summarised in Table 5, when reported). When analysing the total GBI score on an individual level, 98% (172 of 176 patients) reported an improvement in quality of life after Ponto surgery.

**TABLE 5:** Subjective outcome: Quality of life

Ref.	Hearing loss	Device	Average GBI score Scale: −100 to +100	Proportion of patients with total score > 0
33	Conductive, mixed and SSD	Mixed Ponto	Total score: 32.3 General score: 45.5 Social score: 9.72 Physical score: 2.72	98% (49/50)
24	Conductive and mixed	Mixed Ponto	Total score: 39.5	100% (38/38)
35	Conductive, mixed and SSD	Mixed Ponto	Total score: 33.9 General score: 47.5 Social score: 11.0 Physical score: 3.5	98% (56/57)
47	Conductive and mixed and SSD	Mixed Ponto	Total score: 25.3	92% (23/25)
26	Conductive and mixed	Ponto Pro Power	Total score: 32 General score: 49 Social score: 2.8 Physical score: ‐8.3	100% (6/6)

Abbreviations: GBI, Glasgow benefit inventory; SSD, single‐sided deafness.

## 4 DISCUSSION

### 4.1 Summary of the main results

The literature reporting on the Ponto BAHSs was reviewed. In total, data from 1352 patients were included in the review.

It can be concluded that the Ponto system provides an improvement in hearing ability compared to unaided hearing, in terms of both audibility and speech recognition. Based on the review, the average Ponto patient experienced an improvement in hearing of 35 dB. This is the average functional gain or functional benefit reported across studies, with all studies reporting an improvement. From an audiological perspective, the effective gain is more relevant to evaluate and compare system performance since it is not affected by the patients' air‐bone gap.^9^, ^10^ The average effective gain was 6.7 dB across seven studies (98 patients). The effective gain can also be interpreted as the remaining air‐bone gap (compared below).

The data on the implant system confirmed earlier findings and refined the knowledge on complication rates. Major complications (intra‐ or postoperatively) are very rare, with no life‐threatening complications reported in the summarised data. The overall implant survival rate was 98%, with an average follow‐up time of 17 months (0.25‐60 months).

Skin reactions are the most common complication. A Holgers score ≥2 generally warrants treatment, typically local treatment for a Holgers score of 2, with the addition of systemic antibiotic treatment for a Holgers score of 3. Across the studies included in the review, reactions classified as Holgers 2 or higher occurred in 5% of visits and 15% of patients. Thus, from a patient perspective, approximately one in seven patients experienced a skin reaction requiring treatment. Only 0.4% of the patients were reported to have a skin reaction graded as Holgers 4 (the highest grading often requiring removal of the abutment). In addition, revision surgery can become necessary. It can be noted that revision surgery in bone‐anchored cases is generally a minor surgical intervention, for example removing excessive soft tissue. The rate of revision surgery was 3% in the studies that report this outcome.

It can further be concluded that the Ponto system provides consistent improvement in quality of life: 98% (172 of 176 patients) reported an improvement in quality of life after Ponto surgery, as measured with the GBI questionnaire. The average reported GBI score was 32.6 points.

### 4.2 Overall completeness and applicability of evidence

All patient indications were covered in the reviewed data. The proportion of SSD patients in the data set reporting audiological outcomes was 15.4% (59/383). The proportion of SSD patients in the data is representative of European clinical practice but is significantly lower than that in North America. The incidence of severe mixed hearing loss (with BC thresholds larger than 45 dB HL) is underrepresented in the data sets. This might be a reflection of that power, and superpower devices (with a fitting range up to BC thresholds of 65 dB HL^13^) have been developed in later years.

This review was not designed to investigate the difference in results with different generations of implants, sound processors or surgical techniques. Rather, the purpose was to summarise outcomes across those differences over a 10‐year period. Several changes to the surgical techniques and implant designs have occurred in parallel, and to isolate the effects of certain changes, for example those in surgical techniques, only a subset of the studies would be relevant, and preferably randomised control studies^32^ or case series with well‐defined control groups^33^, ^34^ would be available. Similarly, a few studies in this review have investigated the differences in outcomes between implant generations, particularly.^35^

Recent research clearly indicates that despite similar or even the same performance in terms of audibility or speech recognition, patients might experience very important clinical benefits related to listening effort.^36^ Such differences have indeed been shown for bone‐anchored solutions, with reported effects on listening effort,^12^ recall of what has been heard^28^ and learning new words.^27^ However, to enable a systematic review of hearing outcomes, the more commonly reported aided threshold and speech intelligibility measures are summarised here.

Regarding the level of evidence, it can be noted that high‐level evidence is only available for comparisons of different generations of Ponto systems or different surgical techniques. No high‐level evidence studies comparing BAHS with other types of treatments or devices were identified. On the audiological side, the most common design was the intra‐patient crossover design, where a patient compares either two different sound processors or the same sound processor on abutment and softband. The lack of high‐level evidence comparing Ponto to other treatment options is most likely a result of the percutaneous bone‐anchored system being considered a well‐known and described treatment in the research community.^7^

### 4.3 Comparison with other reviews

This review had a different angle than previous reviews of percutaneous BAHS in that it covers both surgical and implant‐related outcomes, as well as audiological outcomes, and further explores a single system only.

The surgical and implant‐related results of this study can be compared to the more general reviews of BAHS^37^, ^38^, ^39^ and with the conclusions of large retrospective studies.^5^, ^40^

In agreement with the literature,^39^ the rate of serious complications was very low (in fact, no cases were found in the data reviewed). The implant survival rate for the BAHS implants found in this review, 98%, compares favourably to that in other studies (corresponding loss rate of 2%). Bezdjian et al^38^ found an overall implant loss rate of 7.3% in a review of 48 articles and 4,116 implants. However, when only including data where age was known and excluding paediatric patients, the survival rate for adults and elderly patients was 97.0% (914/942 implants). The majority of studies in that review had a follow‐up of more than 2 years.

Verheij et al*,* ^37^ in a review of percutaneous bone‐anchored implants installed with tissue preserving techniques (from 2011 and onwards), found an implant survival rate of 97.6%. The follow‐up times of the studies in the review varied between 13 weeks and 5 years.

In a cohort with older, narrow‐diameter implants, Dun et al^5^ reported an implant survival of 91.7% for the whole cohort comprising 1132 implants with a median follow‐up time of 3.6 years. When excluding children and patients with mental retardation from the analysis, the reported implant survival rate was 92.7%. Similarly, Calon et al^40^ reported an overall survival rate of 93.8% for primary implantation (ie excluding patients who underwent re‐implantation after an implant loss) across 550 implants and with a mean follow‐up time of 3.8 years. In addition, primary implant survival rates at different time points after implantation were reported^7^ to be 97% at 1 year, 95% at 5 years and 94% at 10 years postoperatively.

The rate of skin complications according to Holgers classification in the reviewed publications (approximately 15% of patients or 5% of visits) is as good as that reported elsewhere. Dun et al^5^ reported that 4.6% of visits had Holgers ≥ 2 complications across the whole cohort (7415 visits), and the rate decreased to 4.2% after removing the paediatric cohort (6756 visits). Verheij et al^37^ reported a rate of 9.1% of visits with Holgers ≥ 2 across a total of 762 visits (381 implants). The maximum score per implant is the measure the authors favour, as it is more patient‐centric; the results indicate how large of a proportion of patients can expect a skin‐related complication requiring treatment. In addition, the study design/number of visits over the course of a study does not directly affect this score. This variable was reported in a large retrospective study,^41^ reflecting an overlapping cohort with Dun et al.^5^ They reported that 18.4% of the implants led to at least one skin reaction classified as Holgers ≥ 2 (123/669 implants). Finally, skin complications rated on the Holgers scale are a subset of skin‐related complications. Skin overgrowth or other skin‐related issues requiring a (minor) revision surgery to remove soft tissue should also be accounted for to provide a complete picture. The rate of revision surgeries in the reviewed data set is low (3% or 26/773 implants).

This study further allows for comparisons to alternative system types. Two studies recently summarised data on the active transcutaneous device Bonebridge (MedEl)^42^ and passive transcutaneous devices (BAHA^®^ Attract; Cochlear BAS, and Sophono; Medtronics).^43^ Magele et al^42^ performed a meta‐analysis of functional gain for the Bonebridge device and across indications, including conductive, mixed and SSD patients, and reported an average functional gain of 32.7 dB (a statistical meta‐analysis of appraised data resulted in a value of 30.9 dB). The review included patients with SSD and conductive and mixed hearing loss, though with a lower degree of sensorineural losses than the patients included in this review. For transcutaneous systems, Cooper et al^43^ reported an average functional gain of 28.4 ± 2.1 (SD) dB across devices for 136 patients. Unfortunately, effective gain was not reported as part of any of the reviews. The effective gain is not affected by the air‐bone gap and is therefore a better measure for comparing device performance. By analysing a subset of publications from last 3 years (Tables S9 and S10), we calculated an average effective gain of 10.1 dB for active transcutaneous devices and 15.9 dB for passive transcutaneous devices. The effective gain of Ponto was 6.7 dB, which compares favourably with that of passive transcutaneous devices and active transcutaneous devices. This is in agreement with the conclusion by Reinfeldt et al.^44^ who reviewed different types of BC system.

When comparing complications, neither implant loss rates nor Holgers scores can be applied to transcutaneous BC devices. Suggestions have been made for a joint scale,^45^ but it was not reported in any of the studies. Cooper et al^43^ reported “major complications,” defined as those requiring active medical or surgical management or preventing the use of the device completely. Major complications were reported in 5.2% of cases (25/482). Minor complications were reported in 13.1% of the cases reviewed, with a total complication rate of 18.3% (88/482 implants). Applying the same definition to the results of this review, an implant loss would prevent usage completely, whereas Holgers scores would count as a minor complication. It appears that passive transcutaneous devices might have higher complication rates than percutaneous systems. Active transcutaneous devices^42^ show lower rates of both minor and major complications than passive transcutaneous devices.

Quality of life after different interventions was systematically reviewed by Hendry et al^46^ using GBI scores. They concluded that the heterogeneity of the GBI results with BAHS was too high to make a conclusion. That result was not repeated here. The data reviewed gave an average GBI score of 32.6, which can be compared with the following meta‐analysis results^46^: middle ear implants, 16.3 (95% CI: 10.4, 22.1); stapes surgery, 29.9 (95% CI: 21.0, 38.7); and cochlear implants, 38.4 (95% CI: 29.0, 47.9). A total of 98% (172 of 176 patients) reported an improvement in quality of life.

### 4.4 Implications for clinical practice

Bone‐anchored hearing systems and the Ponto system reviewed in this paper consistently improved hearing and quality of life. Complications are rare and typically minor in nature.

When counselling future patients considering their options, the main results of this study indicate that all prospective patients can expect better hearing after surgery and that the improvement is relatively predictable. The risk for any major complication requiring surgery is very low (<5%). One in seven patients can expect a skin complication requiring treatment. Furthermore, 98% of patients reported an improvement in quality of life following the intervention.

## CONFLICT OF INTEREST

Lagerkvist H., Carvalho K., Holmberg M, Petersson U. are under the employment of Oticon Medical. Hultcrantz, M. and Cremers, CW are consultants for Oticon Medical.

## Supporting information

Supplementary Material

## Data Availability

Data sharing is not applicable to this article as no new data were created or analysed in this study.
