# Supplementary material for: Ten years of experience with the Ponto bone‐anchored hearing system—A systematic literature review
Source: Clin Otolaryngol. 2020 May 25;45(5):667–80. doi: 10.1111/coa.13556 (PMC7496709; doi:10.1111/coa.13556)
Supplement: Supplementary file 1 — Supplementary Material [file COA-45-667-s001.docx]

# Digital supplement

**Table 6**. Reported speech recognition thresholds (SRT)

| **Ref.** | **Hearing loss** | **Device** | **Average unaided SRT in quiet [dB HL or SPL]** | **Average aided SRT in quiet [dB HL or dB SPL]** | **Average unaided SRT in noise [dB SNR]** | **Average aided SRT in noise [dB SNR]** |
| --- | --- | --- | --- | --- | --- | --- |
| ^1^ | Mixed | Ponto Pro Power | - | - | - | 0.0 (omni)^†^  6.9 (omni)^‡^ |
|  |  | Ponto 3 SuperPower | - | - | - | -0.8 (omni)^†^  5.2 (omni)^‡^ |
| ^2^ | Conductive and mixed | Ponto Pro | - | - | - | -3.4 (omni)^†^  -1.8 (omni)^§^  -3.9 (dir)^§^ |
| ^3^ | Mixed | Ponto Pro Power | - | 48.1 | - | -1.8 (omni)^†^  -1.7 (dir)^†^  -0.5 (omni)^§^  -2.0 (dir)^§^ |
| ^4^ | Conductive and mixed | Ponto Pro Power | 83.6 | 58.5 | 13.6 | 0.3 (omni)^¶^  -1.0 (dir)^¶^ |
| ^5^ | Conductive and mixed | Mixed Ponto | 74 | 51 | - | - |
| ^6^ | SSD | Ponto Pro and Cochlear BP100 | - | - | 5.4^††^  -1.6^‡‡^ | 3.3^††^  0.6^‡‡^ |
| ^7^ | Conductive and mixed | Ponto Pro | - | - | - | -4.6^†^ |
| ^8^ | Conductive and mixed | Ponto Plus | 64 | 29.5 | - | 0.24 (dir)^§§^ |
| ^9^ | Conductive and mixed, (SSD) | Unknown | - | 20.2 | - | - |
| ^10^ | SSD | Ponto Pro | - | - | 1.7^¶¶^ | 2.4 (omni)^¶¶^  2.4 (split dir)^¶¶^  1.7 (full dir)^¶¶^ |
| ^11^ | Conductive, mixed and SSD | Ponto Pro | - | - | - | -0.7 (omni)^¶, †††^  -9.5 (dir) ^¶, †††^ |
| ^12^ | Conductive and mixed | Ponto Pro Power | 75.7 | 47.8 | - | -5.7^†^ |

^†^ Loudspeaker setup: Speech 0^◦^, Noise 0^◦^

^‡^ Loudspeaker setup: Speech 0^◦^, Noise +90^◦^/-90^◦^/180^◦^
^§^ Loudspeaker setup: Speech 0^◦^, Noise 90^◦^ ipsilateral

^¶^ Loudspeaker setup: Speech 0^◦^, Noise +90^◦^/-90^◦^

^††^ Loudspeaker setup: Speech 0^◦^, Noise at better ear

^‡‡^ Loudspeaker setup: Speech 0^◦^, Noise at poorer ear

^§§^ Loudspeaker setup: Speech 0^◦^, Noise 180^◦^

^¶¶^ Loudspeaker setup: Speech 0^◦^, Noise diffuse

^†††^ Median

**Table 7.** Reported speech recognition scores (SRS)

| **Ref.** | **Hearing loss** | **Device** | **Average unaided SRS in quiet (%)** | **Average aided SRS in quiet (%)** | **Average unaided SRS in noise (%)** | **Average aided SRS in noise (%)** |
| --- | --- | --- | --- | --- | --- | --- |
| ^13^ | Conductive and mixed | Ponto 3 SuperPower | - | - | - | 95.6^†^  93.4^‡^ |
|  |  | Ponto 3 | - | - | - | 94.3^†^  91.0^‡^ |
|  |  | Ponto Pro | - | - | - | 92.3^†^  89.6^‡^ |
| ^14^ | Conductive and mixed | Mixed Ponto | - | 88 | - | - |
| ^6^ | SSD | Ponto Pro and Cochlear BP100 | 95^§^ | 96^§^ | 47^¶^  78^††^ | 62^¶^  76^††^ |
| ^7^ | Conductive and mixed | Ponto Pro | - | - | - | 43.2 (-5 dB SNR)^‡‡^ |
| ^8^ | Conductive and mixed | Ponto Plus | 79 | 78 | - | - |
| ^9^ | Conductive and mixed | Unknown Ponto | - | 94.9 | - | - |
| ^12^ | Conductive and mixed | Ponto Pro Power | - | - | 7.7 (+4 dB SNR)^§§^ | 59.3 (+4 dB SNR)^§§^ |
| ^15^ | Conductive | Ponto Pro | 23.3 | 96.5 | - | - |

^†^ Loudspeaker setup: Speech 0^◦^, Noise +90^◦^/-90^◦^/+150^◦^/-150^◦^, individual SNR

^‡^ Loudspeaker setup: Speech 0^◦^, Noise +90^◦^/-90^◦^/+150^◦^/-150^◦^, individual SNR^†^ - 5 dB

^§^ Loudspeaker setup: Speech at poorer ear

^¶^ Loudspeaker setup: Speech 0^◦^, Noise at better ear

^††^ Loudspeaker setup: Speech 0^◦^, Noise at poorer ear

^‡‡^ Loudspeaker setup: Speech 0^◦^, Noise 0^◦^, -5 dB SNR

^§§^ Loudspeaker setup: Speech 0^◦^, Noise 0^◦^, +4 dB SNR

**Table 8.** Complications/events reported (groups A and B), per publication

| **Author** | **Patients** | **Implants** | **Surgical method^‡‡^** | **Follow-up time (months)** | **Dura exposure** | **CSF leak** | **Drilling into vein or bleeding** | **Holgers 4** | **Revision surgery (skin)** | **Haematoma** | **Abscess** | **Scar hypertrophy** | **Abutment change** | **Abutment removal** | **Pain and numbness outcome reported** |
| --- | --- | --- | --- | --- | --- | --- | --- | --- | --- | --- | --- | --- | --- | --- | --- |
| Calon (2018) ^16^ | 63 | 63 | TP, MIPS | 3 | 1 | 0 | 4 |  | 0 | 2 |  |  | 0 | 0 | X |
| Caruso (2016 ^5^) | 49 | 49 | TP | 9-20 | 13 | 0 | 2 | 0 | 0 |  |  |  | 0 | 1 |  |
| den Besten (2016) ^17^ | 25 | 25 | TP | 6 |  |  |  | 0 | 0 |  |  |  | 0 |  | X |
| Foghsgaard (2014) ^18^ | 20 | 20 | TR | 11.5-15.3, m 12.6 |  |  |  | 0 | 0 |  |  |  | 1 |  |  |
| Hultcrantz (2015)^‡19^ | 2 | 4 | TP | 12 |  |  |  |  |  |  |  |  |  |  |  |
| Johansson (2017) ^20^ | 76 | 77 | MIPS | 5-9.8, m 8.5 | 3 | 1 | 11 | 0 |  |  |  |  |  |  | X |
| Kim (2019) ^21^ | 75 | 75 | MIPS | 0.25-7.29 | 0 | 0 | 3 | 0 | 0 |  |  |  |  |  |  |
| Kruyt (2019) ^22^ | 25 | 25 | TP | 36 |  |  |  | 0 | 2 |  |  |  |  | 2 | X |
| Kruyt (2018) ^22,23^ | 57 | 59 | TR | 36 |  |  |  | 0 | 3 |  |  |  |  | 1 |  |
| Kruyt (2018) ^24^ | 34 | 34 | TR, TP | 7-17, m 15 |  |  |  | 0 | 0 |  |  |  |  |  | X |
| Mowinckel (2016) ^25^ | 24 | 24 | TP | 12 |  |  | 1 | 0 |  |  |  |  | 1 |  | X |
| Muzaffar (2014) ^26^ | 15 | 20 | TR | 0.5-2.25 |  |  |  |  |  |  |  |  |  |  |  |
| Nelissen (2013) ^27,28^ | 31 | 31 | TR | 12.1-25.2 |  |  |  | 0 | 1 |  |  |  | 4 |  |  |
| Reznitsky (2018) ^29^ | 48 | 48 | 50% TR, TP | 48-60 |  |  |  |  |  |  |  |  |  |  |  |
| Sardiwalla (2018) ^30^ | 12 | 12 | Punch | 22 |  |  |  |  | 0 |  |  |  |  |  | X |
| Trobos (2018) ^31^ | 7 | 7 | TP | 12 |  |  |  | 0 |  |  |  |  |  |  |  |
| Wazen (2016) ^32^ | 30 | 30 | TP | 12 |  |  |  | 0 |  |  |  |  |  |  |  |
| Westover (2018) ^33^ | 39 | 39 | TP, MIPS | 11.6-13.3 m 12.4 |  |  |  |  |  |  |  |  |  |  |  |
| Di Giustino (2018) ^34^ | 29 | 30 | 50% TR, TP, MIPS | 12 | 2 | 0 | 4 | 2 |  |  |  |  |  |  |  |
| Dumon (2015) ^35^ | 40 | 40 | 50% TR, Punch | 6-18, m 10.5 |  |  |  | 1 | 1 |  |  |  |  |  |  |
| Goldman (2013) ^36^ | 14 | 15 | TP, Punch | 9-20, m 14.8 |  |  |  | 0 | 0 |  |  |  | 0 |  |  |
| Gordon (2015) ^37^ | 51 | 51 | 67% TR, Punch | 0.25-25 |  |  |  | 0 |  |  |  |  | 1 | 1 |  |
| Hultcrantz (2015) ^38^ | 17 | 17 | TP | 12 |  |  |  | 0 | 1 |  |  |  | 2 | 0 | X |
| Singam (2014) ^39^ | 30 | 30 | TP | 6-42, m 23 |  |  |  | 0 | 3 |  |  |  | 1 |  |  |
| Strijbos (2016) ^40^ | 203 | 211 | TR | 11.2-35.3 |  |  |  |  | 7 |  |  |  | 9 |  |  |
| Wise (2018) ^41^ | 130 | 130 | TR | 6-29, m 16.4 |  |  |  | 0 | 8 |  | 2 | 1 | 8 |  |  |
| # publ. | 0 | 642 |  |  | 19 | 1 | 25 | 3 | 26 | 2 | 2 | 1 | 27 | 5 | 8 |
| # implants |  |  |  |  | 294 | 294 | 318 | 769 | 773 | 63 | 130 | 130 | 666 | 264 | 354 |
| % of implants |  |  |  |  | 6% | 0% | 8% | 0.4% | 3% | 3% | 2% | 1% | 4% | 2% | 2% |

^†^ Control group not included due to duplication reports (Nelissen 2015)
^‡^Paediatric patients only

^§^3y data published (Kruyt 2019), thus not included in meta-analyses

^¶^Investigational non-commercial device excluded

^††^ Randomized controlled trial RCT, Prospective controlled PC, Prospective P, Retrospective controlled RC, Retrospective R, Case report CR
^‡‡^ Tissue reduction TR, Tissue preservation TP, Minimally invasive Ponto surgery MIPS

^§§^ Not including elective removal

**Table 9.** Publications with audiological threshold results for passive and active transcutaneous devices (June 2016 – June 2019)

| **Ref.** | **Author (year)** | **Patients** | **Device(s)** | **Clinical condition** | **Average aided threshold** | **Average functional gain** | **Average calculated effective gain** |
| --- | --- | --- | --- | --- | --- | --- | --- |
| **Publications with passive transcutaneous devices** | | | | | | | |
| ^42^ | den Besten et al (2018) | 54 | Attract with misc. BAHA SP | CHL, MHL | 31 | 21 | 20 |
|  |  |  |  | SSD | 31 | 22 | 23 |
| ^43^ | Carr et al (2017) | 3 | Attract^†^ | CHL, MHL | 27^‡^ | 33^‡^ | - |
| ^44^ | Fan et al (2019) | 11 | Attract^†^ | CHL, MHL | 31 | 35 | - |
| ^45^ | Giannantonio et al (2018) | 20 | Sophono Alpha 2 | CHL | 31 | 41 | **-** |
|  |  |  | Attract with BAHA 5 | CHL | 25 | 43 | **-** |
| ^46^ | Hougaard et al (2017) | 20 | Attract^†^ | CHL, MHL | 41 | 20 | - |
|  |  |  |  | SSD | 35 | 11 | - |
| ^47^ | Magliulo et al (2018) | 14 | Sophono Alpha 1 and Alpha 2 | CHL, MHL | 44 | 27 | 19 |
| ^48^ | Nevoux et al (2018) | 32 | Attract with BAHA 4 | CHL, MHL | 40 | 40 | 9 |
|  |  |  |  | SSD | 37 | 28^‡^ | 16 |
| ^49^ | Pérez-Carbonell et al (2017) | 9 | Attract with BP100 | CHL | 36 | 26 | 14 |
| ^50^ | Polonenko et al (2016) | 9 | Sophono Alpha 2 | CHL, MHL | 20 | 48 | 11 |
| **Publications with active transcutaneous devices** | | | | | | | |
| ^51^ | Bravo-Torres et al (2018) | 15 | Bonebridge^†^ | CHL | 25 | 41^‡^ | - |
| ^52^ | Carnevale et al (2018) | 26 | Bonebridge^†^ | CHL, MHL | 35 | 33 | 4 |
| ^53^ | Chan et al (2019) | 10 | Bonebridge^†^ | CHL | 29 | 35 | 21 |
| ^54^ | Ihler et al (2016) | 8 | Bonebridge Amadé | CHL, MHL | 24 | 29 | 5 |
| ^55^ | Kulasegarah et al (2018) | 10 | Bonebridge^†^ | CHL | 26 | 36 | 15 |
| ^56^ | Lin et al (2019) | 20 | Bonebridge Amadé | CHL | 38 | 27 | - |
| ^57^ | Ngui & Tang (2018) | 6 | Bonebridge^†^ | CHL | 26 | 46 | 13 |
| ^58^ | Rader et al (2018) | 61 | Bonebridge^†^ | CHL, MHL | 33 | 30 | - |
| ^59^ | Ratuszniak et al (2018) | 11 | Bonebridge^†^ | CHL, MHL | 32 | 30 | 15 |
| ^60^ | Salcher et al (2017) | 10 | Bonebridge^†^ | SSD | 28 | 36^‡^ | 17 |
| ^61^ | Schmerber et al (2017) | 13 | Bonebridge Amadé | CHL, MHL | 25 | 26 | 8 |
| ^62^ | Skarzynski et al (2019) | 21 | Bonebridge^†^ | CHL, MHL | 33 | 28 | 12 |
| ^63^ | Tang et al (2018) | 20 | Bonebridge^†^ | CHL, MHL, SSD | 27 | 55^‡^ | -5 |
| ^64^ | Vyskocil et al (2017) | 5 | Bonebridge^†^ | CHL | - | 26 | - |
| ^65^ | Weiss et al (2017) | 18 | Bonebridge^†^ | CHL, MHL | - | 29 | - |
| ^66^ | Wimmer et al (2019) | 28 | Bonebridge^†^ | MHL, SSD | 29 | 29 | - |
| ^67^ | Zernotti et al (2019) | 14 | Bonebridge^†^ | CHL | 19 | 47 | 7 |

^†^ Undefined sound processor model

^‡^ Value derived from graph in the publication

**Table 10**. Summary of hearing loss and aided thresholds (average values reported per study were used for the calculations) for passive and active transcutaneous systems

| **Outcome** | **Number of studies reporting the variable** | | **Number of patients** | | **Value ± SD (min, max)** | |
| --- | --- | --- | --- | --- | --- | --- |
|  | **Passive** | **Active** | **Passive** | **Active** | **Passive** | **Active** |
| Aided threshold | 9 | 15 | 172 | 273 | 32.8 dB HL ± 6.7 (20.4, 44.2) | 28.7 dB HL ± 4.9 (19.3, 38.0) |
| Functional gain | 9 | 17 | 172 | 296 | 30.5 dB ± 10.0 (14.8, 47.6) | 34.3 dB HL ± 8.5 (25.7, 55.0) |
| Effective gain | 5 | 11 | 118 | 149 | 15.9 dB ± 5.0 (9.0, 22.7) | 10.1 dB HL ± 7.3 (-5.0, 21.0) |

## Reference list for Digital supplement

1. Bosman AJ, Kruyt IJ, Mylanus EA, Hol MK, Snik AF. Evaluation of an Abutment-level SuperPower Sound Processor for Bone-Anchored Hearing. *Clinical otolaryngology : official journal of ENT-UK ; official journal of Netherlands Society for Oto-Rhino-Laryngology & Cervico-Facial Surgery.* 2018.

2. Bosman A, Hol M, Snik A, Mylanus E. Evaluating Oticon Medical Ponto Plus and Ponto Pro Sound Processor. In. <http://www.audiologyonline.com/articles/evaluating-oticon-medical-ponto-plus-12616>, 2014.

3. Bosman AJ, Snik AF, Hol MK, Mylanus EA. Evaluation of a new powerful bone-anchored hearing system: a comparison study. *Journal of the American Academy of Audiology.* 2013;24(6):505-513.

4. Busch S, Giere T, Lenarz T, Maier H. Comparison of audiologic results and patient satisfaction for two osseointegrated bone conduction devices: results of a prospective study. *Otol Neurotol.* 2015;36(5):842-848.

5. Caruso A, Giannuzzi AL, Sozzi V, Sanna M. Bone anchored hearing implants without skin thinning: the Gruppo Otologico surgical and audiological experience. *Eur Arch Otorhinolaryngol.* 2017;274(2):695-700.

6. Finbow J, Bance M, Aiken S, Gulliver M, Verge J, Caissie R. A Comparison Between Wireless CROS and Bone-anchored Hearing Devices for Single-sided Deafness: A Pilot Study. *Otol Neurotol.* 2015;36(5):819-825.

7. Hill-Feltham P, Roberts SA, Gladdis R. Digital processing technology for bone-anchored hearing aids: randomised comparison of two devices in hearing aid users with mixed or conductive hearing loss. *The Journal of laryngology and otology.* 2014;128(2):119-127.

8. Kara A, Guven M, Sinan Yilmaz M, et al. Comparison of two different bone anchored hearing instruments: Baha-5 vs Ponto-plus. *Acta Otolaryngol.* 2019;139(6):517-521.

9. Nelissen RC, Mylanus EA, Kunst HP, Pennings RJ, Snik AF, Hol MK. A new bone-anchored hearing implant: short-term retrospective data on implant survival and subjective benefit. *Eur Arch Otorhinolaryngol.* 2013;270(12):3019-3025.

10. Oeding K, Valente M. The effectiveness of the directional microphone in the Oticon Medical Ponto Pro in participants with unilateral sensorineural hearing loss. *Journal of the American Academy of Audiology.* 2013;24(8):701-713.

11. Olsen SO, Glad H, Nielsen LH. Comparison of two bone anchored hearing instruments: BP100 and Ponto Pro. *International journal of audiology.* 2011;50(12):920-928.

12. Rigato C, Reinfeldt S, Hakansson B, Jansson KJ, Hol MK, Eeg-Olofsson M. Audiometric Comparison Between the First Patients With the Transcutaneous Bone Conduction Implant and Matched Percutaneous Bone Anchored Hearing Device Users. *Otol Neurotol.* 2016;37(9):1381-1387.

13. Bianchi F, Wendt D, Wassard C, et al. Benefit of Higher Maximum Force Output on Listening Effort in Bone-Anchored Hearing System Users: A Pupillometry Study. *Ear Hear.* 2019.

14. Celikgun B, Kalcioglu MT. Assessment of discrimination ability in ipsilateral and contralateral ears with a unilateral bone-anchored hearing system. *Ear Nose Throat J.* 2017;96(8):297-310.

15. Wang Y, Fan X, Wang P, Fan Y, Chen X. Hearing improvement with softband and implanted bone-anchored hearing devices and modified implantation surgery in patients with bilateral microtia-atresia. *International journal of pediatric otorhinolaryngology.* 2018;104:120-125.

16. Calon TGA, Johansson ML, de Bruijn AJG, et al. Minimally Invasive Ponto Surgery Versus the Linear Incision Technique With Soft Tissue Preservation for Bone Conduction Hearing Implants: A Multicenter Randomized Controlled Trial. *Otol Neurotol.* 2018;39(7):882-893.

17. den Besten CA, Bosman AJ, Nelissen RC, Mylanus EA, Hol MK. Controlled Clinical Trial on Bone-anchored Hearing Implants and a Surgical Technique With Soft-tissue Preservation. *Otol Neurotol.* 2016;37(5):504-512.

18. Foghsgaard S, Caye-Thomasen P. A new wide-diameter bone-anchored hearing implant-prospective 1-year data on complications, implant stability, and survival. *Otol Neurotol.* 2014;35(7):1238-1241.

19. Hultcrantz M, Lanis A. Stability testing after osseointegrated implant surgery without skin thinning in children: case reports after abutment loss. *Otol Neurotol.* 2014;35(6):1102-1104.

20. Johansson ML, Stokroos RJ, Banga R, et al. Short-term results from seventy-six patients receiving a bone-anchored hearing implant installed with a novel minimally invasive surgery technique. *Clinical otolaryngology : official journal of ENT-UK ; official journal of Netherlands Society for Oto-Rhino-Laryngology & Cervico-Facial Surgery.* 2017;42(5):1043-1048.

21. Kim HHS, Kari E, Copeland BJ, et al. Standardization of the Punch Technique for the Implantation of Bone Anchored Auditory Devices: Evaluation of the MIPS Surgical Set. *Otol Neurotol.* 2019;40(6):e631-e635.

22. Kruyt IJ, Kok H, Bosman A, Nelissen RC, Mylanus EAM, Hol MKS. Three-Year Clinical and Audiological Outcomes of Percutaneous Implants for Bone Conduction Devices: Comparison Between Tissue Preservation Technique and Tissue Reduction Technique. *Otol Neurotol.* 2019;40(3):335-343.

23. Kruyt IJ, Nelissen RC, Mylanus EAM, Hol MKS. Three-year Outcomes of a Randomized Controlled Trial Comparing a 4.5-mm-Wide to a 3.75-mm-Wide Titanium Implant for Bone Conduction Hearing. *Otol Neurotol.* 2018.

24. Kruyt IJ, Banga R, Banerjee A, Mylanus EAM, Hol MKS. Clinical evaluation of a new laser-ablated titanium implant for bone-anchored hearing in 34 patients: 1-year experience. *Clinical otolaryngology : official journal of ENT-UK ; official journal of Netherlands Society for Oto-Rhino-Laryngology & Cervico-Facial Surgery.* 2018.

25. Mowinckel MS, Moller MN, Wielandt KN, Foghsgaard S. Clinical Outcome of a Wide-diameter Bone-anchored Hearing Implant and a Surgical Technique With Tissue Preservation. *Otol Neurotol.* 2016;37(4):374-379.

26. Muzaffar SJ, Coulson CJ, Burrell S, Reid AP. Initial experience of a rapid-insertion bone-anchored hearing system: series of 20 consecutive implants. *The Journal of laryngology and otology.* 2014:1-5.

27. Nelissen RC, den Besten CA, Mylanus EA, Hol MK. Stability, survival, and tolerability of a 4.5-mm-wide bone-anchored hearing implant: 6-month data from a randomized controlled clinical trial. *Eur Arch Otorhinolaryngol.* 2016;273(1):105-111.

28. Nelissen RC, den Besten CA, Mylanus EA, Hol MK. Erratum to: Stability, survival, and tolerability of a 4.5-mm-wide bone-anchored hearing implant: 6-month data from a randomized controlled clinical trial. *Eur Arch Otorhinolaryngol.* 2016;273(1):113-114.

29. Reznitsky M, Wielandt K, Foghsgaard S. Wide diameter bone-anchored hearing system implants: a comparison of long-term follow-up data between tissue reduction and tissue preservation techniques. *Eur Arch Otorhinolaryngol.* 2018.

30. Sardiwalla Y, Jufas N, Morris DP. Long term follow-up demonstrating stability and patient satisfaction of minimally invasive punch technique for percutaneous bone anchored hearing devices. *Journal of otolaryngology - head & neck surgery = Le Journal d'oto-rhino-laryngologie et de chirurgie cervico-faciale.* 2018;47(1):71.

31. Trobos M, Johansson ML, Jonhede S, et al. The clinical outcome and microbiological profile of bone-anchored hearing systems (BAHS) with different abutment topographies: a prospective pilot study. *Eur Arch Otorhinolaryngol.* 2018;275(6):1395-1408.

32. Wazen JJ, Babu S, Daugherty J, Metrailer A. Three-week loading of the 4.5mm wide titanium implant in bone anchored hearing systems. *American journal of otolaryngology.* 2016;37(2):132-135.

33. Westover L, Faulkner G, Hodgetts W, Kamal F, Lou E, Raboud D. Longitudinal Evaluation of Bone-Anchored Hearing Aid Implant Stability Using the Advanced System for Implant Stability Testing (ASIST). *Otol Neurotol.* 2018;39(6):e489-e495.

34. Di Giustino F, Vannucchi P, Pecci R, Mengucci A, Santimone R, Giannoni B. Bone-anchored hearing implant surgery: our experience with linear incision and punch techniques. *Acta Otorhinolaryngol Ital.* 2018;38(3):257-263.

35. Dumon T, Medina M, Sperling NM. Punch and Drill: Implantation of Bone Anchored Hearing Device Through a Minimal Skin Punch Incision Versus Implantation With Dermatome and Soft Tissue Reduction. *Ann Otol Rhinol Laryngol.* 2016;125(3):199-206.

36. Goldman RA, Georgolios A, Shaia WT. The punch method for bone-anchored hearing aid placement. *Otolaryngology--head and neck surgery : official journal of American Academy of Otolaryngology-Head and Neck Surgery.* 2013;148(5):878-880.

37. Gordon SA, Coelho DH. Minimally Invasive Surgery for Osseointegrated Auditory Implants: A Comparison of Linear versus Punch Techniques. *Otolaryngology--head and neck surgery : official journal of American Academy of Otolaryngology-Head and Neck Surgery.* 2015;152(6):1089-1093.

38. Hultcrantz M. Stability Testing of a Wide Bone-Anchored Device after Surgery without Skin Thinning. *Biomed Res Int.* 2015;2015:853072.

39. Singam S, Williams R, Saxby C, Houlihan FP. Percutaneous bone-anchored hearing implant surgery without soft-tissue reduction: up to 42 months of follow-up. *Otol Neurotol.* 2014;35(9):1596-1600.

40. Strijbos RM, den Besten CA, Mylanus EA, Hol MK. Percutaneous bone-anchored hearing implant surgery: inside or outside the line of incision? *Eur Arch Otorhinolaryngol.* 2016;273(11):3713-3722.

41. Wise SR, LaRouere JS, Bojrab DI, LaRouere MJ. Comparison Study of Percutaneous Osseointegrated Bone Conduction Device Complications When Using the 9 mm Abutment Versus 6 mm Abutment at Initial Implantation. *Otol Neurotol.* 2018;39(4):451-457.

42. den Besten CA, Monksfield P, Bosman A, et al. Audiological and clinical outcomes of a transcutaneous bone conduction hearing implant: Six-month results from a multicentre study. *Clinical otolaryngology : official journal of ENT-UK ; official journal of Netherlands Society for Oto-Rhino-Laryngology & Cervico-Facial Surgery.* 2018.

43. Carr SD, Bruce IA, Jones D, Ray J. Outcomes following conversion of a percutaneous to a transcutaneous bone conduction device in eight children. *Clinical otolaryngology : official journal of ENT-UK ; official journal of Netherlands Society for Oto-Rhino-Laryngology & Cervico-Facial Surgery.* 2017;42(4):917-920.

44. Fan X, Chen Y, Niu X, Wang Y, Fan Y, Chen X. Outcomes of the Baha Attract System combined with auricle reconstruction in mandarin speaking patients with bilateral microtia-atresia. *Acta Otolaryngol.* 2019:1-7.

45. Giannantonio S, Scorpecci A, Pacifico C, Marsella P. A functional and anatomical comparison between two passive transcutaneous bone conduction implants in children. *International journal of pediatric otorhinolaryngology.* 2018;108:202-207.

46. Hougaard DD, Boldsen SK, Jensen AM, Hansen S, Thomassen PC. A multicenter study on objective and subjective benefits with a transcutaneous bone-anchored hearing aid device: first Nordic results. *Eur Arch Otorhinolaryngol.* 2017;274(8):3011-3019.

47. Magliulo G, Iannella G, De Vincentiis M, et al. Transcutaneous bone conductive implants in patients with conductive/mixed hearing loss: audiological outcomes in noise condition. *Acta Otolaryngol.* 2018:1-8.

48. Nevoux J, Coudert C, Boulet M, et al. Transcutaneous Baha Attract system: long-term outcomes of the French multicenter study. *Clinical otolaryngology : official journal of ENT-UK ; official journal of Netherlands Society for Oto-Rhino-Laryngology & Cervico-Facial Surgery.* 2018.

49. Perez-Carbonell T, Pla-Gil I, Redondo-Martinez J, Morant-Ventura A, Garcia-Callejo FJ, Marco-Algarra J. Audiologic and subjective evaluation of Baha((R)) Attract device. *Acta otorrinolaringologica espanola.* 2017;68(6):344-348.

50. Polonenko MJ, Carinci L, Gordon KA, Papsin BC, Cushing SL. Hearing Benefit and Rated Satisfaction in Children with Unilateral Conductive Hearing Loss Using a Transcutaneous Magnetic-Coupled Bone-Conduction Hearing Aid. *Journal of the American Academy of Audiology.* 2016;27(10):790-804.

51. Bravo-Torres S, Der-Mussa C, Fuentes-Lopez E. Active transcutaneous bone conduction implant: audiological results in paediatric patients with bilateral microtia associated with external auditory canal atresia. *International journal of audiology.* 2018;57(1):53-60.

52. Carnevale C, Til-Perez G, Arancibia-Tagle DJ, Tomas-Barberan MD, Sarria-Echegaray PL. Hearing outcomes of the active bone conduction system Bonebridge((R)) in conductive or mixed hearing loss. *Acta otorrinolaringologica espanola.* 2018.

53. Chan KC, Wallace CG, Wai-Yee Ho V, Wu CM, Chen HY, Chen ZC. Simultaneous auricular reconstruction and transcutaneous bone conduction device implantation in patients with microtia. *J Formos Med Assoc.* 2019.

54. Ihler F, Blum J, Berger MU, Weiss BG, Welz C, Canis M. The Prediction of Speech Recognition in Noise With a Semi-Implantable Bone Conduction Hearing System by External Bone Conduction Stimulation With Headband: A Prospective Study. *Trends Hear.* 2016;20.

55. Kulasegarah J, Burgess H, Neeff M, Brown CRS. Comparing audiological outcomes between the Bonebridge and bone conduction hearing aid on a hard test band: Our experience in children with atresia and microtia. *International journal of pediatric otorhinolaryngology.* 2018;107:176-182.

56. Lin J, Chen S, Zhang H, et al. Application of Implantable Hearing Aids and Bone Conduction Implant System in patients with bilateral congenital deformation of the external and middle ear. *International journal of pediatric otorhinolaryngology.* 2019;119:89-95.

57. Ngui LX, Tang IP. Bonebridge transcutaneous bone conduction implant in children with congenital aural atresia: surgical and audiological outcomes. *The Journal of laryngology and otology.* 2018:1-5.

58. Rader T, Stover T, Lenarz T, et al. Retrospective Analysis of Hearing-Impaired Adult Patients Treated With an Active Transcutaneous Bone Conduction Implant. *Otol Neurotol.* 2018;39(7):874-881.

59. Ratuszniak A, Skarzynski PH, Gos E, Skarzynski H. The Bonebridge implant in older children and adolescents with mixed or conductive hearing loss: Audiological outcomes. *International journal of pediatric otorhinolaryngology.* 2018;118:97-102.

60. Salcher R, Zimmermann D, Giere T, Lenarz T, Maier H. Audiological Results in SSD With an Active Transcutaneous Bone Conduction Implant at a Retrosigmoidal Position. *Otol Neurotol.* 2017;38(5):642-647.

61. Schmerber S, Deguine O, Marx M, et al. Safety and effectiveness of the Bonebridge transcutaneous active direct-drive bone-conduction hearing implant at 1-year device use. *Eur Arch Otorhinolaryngol.* 2017;274(4):1835-1851.

62. Skarzynski PH, Ratuszniak A, Krol B, et al. The Bonebridge in Adults with Mixed and Conductive Hearing Loss: Audiological and Quality of Life Outcomes. *Audiology & neuro-otology.* 2019:1-10.

63. Tang IP, Ling XN, Prepageran N. A review of surgical and audiological outcomes of bonebridge at tertiary centres in Malaysia. *Med J Malaysia.* 2018;73(5):276-280.

64. Vyskocil E, Liepins R, Kaider A, Blineder M, Hamzavi S. Sound Localization in Patients With Congenital Unilateral Conductive Hearing Loss With a Transcutaneous Bone Conduction Implant. *Otol Neurotol.* 2017;38(3):318-324.

65. Weiss R, Leinung M, Baumann U, Weissgerber T, Rader T, Stover T. Improvement of speech perception in quiet and in noise without decreasing localization abilities with the bone conduction device Bonebridge. *Eur Arch Otorhinolaryngol.* 2017;274(5):2107-2115.

66. Wimmer W, von Werdt M, Mantokoudis G, Anschuetz L, Kompis M, Caversaccio M. Outcome prediction for Bonebridge candidates based on audiological indication criteria. *Auris Nasus Larynx.* 2019.

67. Zernotti ME, Chiaraviglio MM, Mauricio SB, Tabernero PA, Zernotti M, Di Gregorio MF. Audiological outcomes in patients with congenital aural atresia implanted with transcutaneous active bone conduction hearing implant. *International journal of pediatric otorhinolaryngology.* 2019;119:54-58.
